# Selections and Abstracts

**Published:** 1914-09

**Authors:** 


### SELECTIONS AND ABSTRACTS

### THE AMERICAN ASSOCIATION FOR STUDY AND PREVENTION OF INFANT MORTALITY.

Boston, November 12-14,1914.

The Fifth Annual Meeting of the Association will be held in Boston November 12-14. The program will include sessions arranged by the Committees on Nursing and Social Work, Pediatrics, Vital and Social Statistics, Obstetrics, and Public School Education. The subjects to be discussed will include:

Prenatal Care

The Need for Inreased and Improved Maternity Hospital Service

Institutional Mortality

Continuation Schools of Home-Making.

The Session on Nursing and Social Work and the Joint Session on Pediatrics and Vital and Social Statistics will be held at the Harvard Medical School. All other Sessions will be held at the Copley Plaza Hotel.

Special clinics will be held on the opening day of the meeting at the Harvard Medical School and elsewhere, the exact time and place to be announced later.

An exhibit will be held in connection with the meeting.

The Chairman of the Committee on Local Arrangements is Dr. Hugh Cabot, 87 Marlboro Street, Boston, Mass.

Further information or circulars in regard to the work of the Association can be secured from the Executive Secretary, 1211 Cathedral Street, Baltimore, Md.

---

"Owing to the depressed conditions throughout the country, the clinical meeting of the Georgia Surgeons' Club has been postponed from November to some time in the spring after the unsettled conditions have been adjusted."

---

### CONSERVATION OF VISION.

By Frank Allport, M. D., Chicago.

Societies for the conservation of vision have been formed in various portions of the United States. They exist under different conditions. The membership in some is medical and in others, lay and medical. In Pennsylvania, Indiana, Missouri, etc., the organization exists as a committee of the State medical society; in New York, Illinois, etc., independent associations have been formed; in Ohio the "commission," as it is called, is a part of the State government. There is also a National association, independent in its nature, and mixed in its membership. Some of these associations, as in New York, Massachusetts, Maryland, Ohio, etc., are doing energetic work, while others are inactive and almost useless.

At the 1913 meeting of the American Medical Association the Council on Health and Public Instruction appointed a Committee on Conservation of Vision consisting of Dr. E. M. Alger, of New York; Dr. W. E. Bruner, of Cleveland; Dr. H. D. Bruns, of New Orleans; Dr. J. J. Carroll, of Baltimore; Dr. E. C. Ellett, of Memphis; Dr. Harold Gifford, of Omaha;

Dr. Vard Hulen, of San Francisco; Dr. W. B. Lancaster, of Boston; Dr. F. Park Lewis, of Buffalo; Dr. William C. Posey, of Philadelphia; Dr. W. H. Wilder, of Chicago; Dr. Casey A. Wood, of Chicago; Dr. Hiram Woods, of Baltimore; and Dr. Frank Allport, of Chicago; of this committee I was appointed chairman. The object of the committee was to produce interest and action in conserving vision, and to endeavor to concentrate under the auspices of the American Medical Association activities calculated to preserve the sight of this and coming generations.

We began our work by using the machinery of the American Medical Association. The Council on Health and Public Instruction sends out each week a sheet, called the Press Bulletin, free to nearly six thousand newspapers, and the printed matter upon its face can be used by the papers as editorial or news matter. The printed matter consists of short, plainly written articles on health topics. They are unsigned. The council employs clipping bureaus, and through them it is ascertained that these articles are extensively used. They are shaping public thought along medical lines in this country. The conservation of vision committee has an apropriate article in the Press Bulletin each week, and its articles on The Eyes, and the Movies, Face Powder and the Eyes, Cross Eyes, Illumination in Homes, Offices, Schools, etc., Golf Accidents to the Eyes, What is a Cataract?, etc., have been extensively copied all over the country. The committee feels that this is a most important part of its work, as it reaches so many people and is under direct control.

The next work of the committee was to write, print and circulate twenty pamphlets on popular eye topics. These are called the Conservation of Vision pamphlets and are numbered from one to twenty. They are handsomely gotten up in uniform style. The authors' names are given. They are short and plainly written, so that nonmedical people can easily understand them. They are well illustrated. They sell for five cents a copy, or one hundred copies for $3.50. They are also freely given away on proper application either to me, as chairman of the committee, or to Dr. F. R. Green, Council on Health and Public Instruction, American Medical Association, 535 North Dearborn street, Chicago. Sets of these pamphlets have been sent to public libraries, women's clubs, teachers' institutes, State legislatures, health boards, etc., all over the United States, and the sale and the distribution have been extensive. Some of the pamphlets are already in their third edition. Arrangements are being made to distribute Doctor Carroll's Pamphlet on the Eyes of Transportation Employees to every railroad surgeon in the United States. I give here a list of the pamphlets:

I. Schoolchildren's Eyes, by Dr. Frank Allport, Chicago.

II. Industrial and Household Accidents to the Eye, by Dr. Harold Gifford, Omaha.

III. Wearing Glasses, by Dr. W. B. Lancaster, Boston.

IV. The Relation of Illumination to Visual Efficiency, by Dr. Ellice M. Alger, New York.

V. Trachoma in Eastern Kentucky, by Dr. J. A. Stucky, Lexington, Ky.

VI. Autointoxication and the Eye, by Dr. H. D. Bruns, New Orleans.

VII. Eye Strain, by Dr. Hiram Woods, Baltimore.

VIII. Lenses and Refraction, by Dr. Frank Allport, Chicago.

IX. The Eye and Its Functions, by Dr. Frank Allport, Chicago.

X. Care of the Eyes, by Dr. Frank Allport, Chicago.

XI. Infant Blindness, or Ophthalmia Neonatorum, by Dr. F. Park Lewis, Buffalo, N. Y.

XII. Ordinary Eye Diseases, by Dr. L. W. Dean, Iowa City, Iowa.

XIII. Usual and Unusual Eye Accidents, by Dr. E. C. Ellett, Memphis, Tenn.

XIV. The Eyes of Transportation Employees, by Dr. J. J. Carroll, Baltimore.

XV. Ocular Hygiene in Schools, by Dr. S. D. Risley, Philadelphia.

XVI. Whisky, Tobacco and Drugs and the Eye, by Dr. Edward Jackson, Denver.

XVII. The Oculist and the Optician, by Dr. Melville Black, Denver.

XVIII. Preparation for Blindness, by Dr. F. Park Lewis, Buffalo.

XIX. What to Do for Blind Children, by Dr. F. Park Lewis, Buffalo.

XX. Blindness from Wood Alcohol, by Dr. Casey Wood, Chicago.

I should like to suggest that these are excellent pamphlets for doctors to keep in their offices and on their reading tables for distribution and education. I am constantly giving away these pamphlets to patients who desire them. Some people wish information on one subject and others desire knowledge along other lines. The pamphlet topics are sufficiently varied to fill the wants of most people. Besides, it often happens that an oculist desires to instruct a certain patient on a particular subject. Almost all eye subjects are mentioned in these pamphlets and are plainly and understandingly discussed, and it would be far easier to give such patients an appropriate pamphlet or two than to take the time to explain the subject personally.

It seemed desirable to the committee to create a national sentiment in favor of conservation of vision, and for this purpose it was determined that lectures on this subject should be delivered in each State. A lecture bureau manager was appointed in each State who was willing to superintend the work. Where a State organization for the conservation of vision existed, one of its officers was selected to carry on the work; but where no such State organization existed, a prominent, energetic, and willing oculist was selected. The plan was about as follows:

The State manager, as he is called, must enlist the assistance of all the oculists he could to assist him in his work, acting, wherever possible, in harmony with local and State medical societies, both ophthalmological and general. Prominent and energetic oculists must be found who should be willing to lecture on the conservation of vision on invitation. Such lecturers should reside in different portions of the State, so that long and expensive journeys might not be necessary. The State manager should correspond with local medical societies, women's clubs, teachers' institutes, boards of health and education, etc., and secure invitations for a lecturer to go to different cities to talk on the conservation of vision. Lectures should, if possible, be arranged for one month in advance, to give time for preparation and necessary ethical advertising. The lecturer's expenses should, if possible, be paid for by those issuing the invitation. The lecture should last about one hour and should be given in plain and unscientific language. A discussion should follow. In order to make these lectures easy to deliver, the committee prepared a box of about thirty colored and uncolored stereopticon slides and sent them to each State manager, to be loaned to his associates in the work whenever a lecture was to be delivered. These slides were securely packed in a strong box and locked with a Yale key. The box could be shipped from place to place by express, the key going to its destination by mail. In addition, a pamphlet was prepared, entitled A Plan of Campaign for the Conservation of Vision. This pamphlet contained a full description of the plan and what it was proposed to accomplish. A kind of skeleton lecture was included in this pamphlet, suggesting subjects, etc., to be referred to in the lecture. These pamphlets were freely dispensed to all State managers, who distributed them to their associates in the work. Beside this, each lecturer was placed on the mailing list for the Press Bulletin, to which reference has already been made, so that he could see the new articles as they came out each week, and perhaps use them in his lectures. Each lecturer also received a complete set of the conservation of vision pamphlets, as it was thought that these would greatly assist him in preparing his lectures. Quantities of the Vision Charts for Schools, were sent into each State so that the lecturers could have all they desired. While these lectures were intended to cover all avenues of vision sonservation, it was especially hoped that they would result in the use of the Crede treatment of the eyes of all new-born babes; in the lessening of shop accidents; and in the annual and systematic examination of all school children's eyes by school teachers; for it is reasonably certain that if these three procedures could be universally adopted, eighty per cent of all blindness could be eliminated from this country. The Crede treatment for newborn infants, the providing and using of goggles and other shop safety devices for eyes, and the annual, systematic examination of school children's eyes, ears, noses and throats by school teachers in the United States could be accomplished at an annual cost not to exceed $250,000. It costs $15,000,000 a year to care for the dependent blind in this country alone, to say nothing of those children more or less incapacitated by defective or diseased ears, noses and throats. Inasmuch as the examination of ears, noses and throats is provided for in the questions annexed to the vision chart, for the teachers' use, and as abnormal conditions of these organs can be investigated easily and speedily, at the same time that the eyes are examined, and, as the benefits to be achieved by this addition are enormous, it is earnestly recommended that teachers will include the ears, noses and throats in their investigations. Emphasis should be laid on several points connected with these annual, systematic examinations of school children's eyes, etc., by school teachers:

1. The examinations are simple and require no medical education.

2. Teachers are not expected to make diagnoses. They merely ascertain the fact through the questions, that something is wrong, and leave the rest to the doctor selected by the family for consultation.

3. A child can be easily examined in five minutes, and each teacher should examine the children attending her own room. By subdividing the work in this way, all the children in any city of any size can be easily examined in one day. A definite day in the early fall of each year should be set aside for these tests in all cities.

4. These tests not only benefit the children by leading to a correction of their eye, ear, nose and throat defects, but the correction of these defects benefits the teachers, because such corrections usually add materially to the intellectual and moral character of the children, thus rendering them much easier to teach, and more pliable to discipline. For either selfish or unselfish reasons teachers should therefore be glad to do this work without grumbling.

5. There is no objection to these examinations being made by doctors or school nurses. This, however, would cost large sums of money and boards of education and health are never allowed enough money for even ordinary purposes. This is a fact which might as well be frankly recognized and acted upon. Let teachers therefore devote one day in the year to this work and get it done. They are easily capable of doing it; it takes no extra time; it is a benefit to themselves as well as to the children; it costs practically nothing. Therefore let it be done.

The question may be pertinently asked, What has been the result of the State lectures? This has varied greatly in dicerent States. No man was compelled to accept the position of State lecture bureau manager; the acceptance was voluntary and was decided after the work and ideals were thoroughly explained. After a promise of conscientious work was given, the box of slides and the printed matter were sent, which represented an outlay of about twenty-five dollars for each State. In some States this (at least, so far as results are concerned) has ended the matter. So far as I know, no work whatever has been done in some States, no lectures have been delivered, and nothing has been accomplished. My letters have been unanswered, my appeals have been unheeded. Perhaps a letter has been finally, at the end of the season, received, informing me that I do not understand conditions in that particular State, or that sickness or business or moving or bad weather or some equally important reason has prevented taking up the lecture plan. Of course, after a gentleman accepts a position of this kind and agrees to do the work, I felt I had a right to depend upon his co-operation, and having once appointed him, the State was left in his charge and I felt that I had no right to interfere. It has been therefore most discouraging to me at the end of the season, when it was about time for me to make my report to the Council on Health and Public Instruction, and when it was too late to change State managers, to realize that no real work had been accomplished in not a few States. In some States, the work, for one reason or another, has been delayed, but still work has been done, as many lecture engagements have been made for next fall with medical societies, teachers' institutes, Chautauqua meetings, women's clubs, educational conventions, etc. But I rejoice to say that in most of the States, honest, conscientious work has been accomplished and excellent results have been achieved. The results are, of course, not all that could be desired, but still, considering that this is the first year of the committee's existence, I should be ungrateful indeed if I did not feel gratified and much obliged to those gentlemen who have given this project their time, their labor, and their means. I beg to present, toward the end of this article, a tabulated report of this wosk up to the time of giving this narrative into the hands of the printer.

The question as to the future of this work now remains to be discussed. I would rceommend:

1. The continuance of a weekly article on the eye for the Press Bulletin of the Council on Health and Public Instruction.

2. A few more conservation of vision pamphlets on interesting subjects and the increased circulation of these pamphlets.

3. The continuation of the conservation of vision lectures in the various States. Many more of these lectures should be delivered, and the State managers should be changed where the present managers have not shown a reasonable interest in the work. From twenty to twenty-five new slides for the stereopticon should be made, that will more fully illustrate the subject.

4. I was as first in favor of encouraging the formation of State societies for the conservation of vision, these societies to possess complete self government, but all of them to declare themselves in affiliation with the Committee on Conservation of Vision of the Council on Health and Public Instruction of the American Medical Association, by a slender thread of connection. I was also in favor of asking those States already possessing State organizations to join with us in the same sort of affiliation. I was also in favor of proposing an annual meeting of this affiliated organization and the formation of a National body of this nature for the conservation of vision. Time and experience have, however, considerably modified my views, and I now believe that other plans are better.

In the first place, there is already a National society of this nature. It is, it is true, highly inactive, but still it exists and should, I believe, be encouraged to an awakening activity. In the second place, local conditions in many of the States are not conducive of friendly assistance. However well meant such assistance may be, it will in some States be regarded as meddlesome interference, and as an effort to rob existing organizations, whether active or lethargic, of some credit which, it is felt, should remain with the local organization and not be shared with even a well intending interloper. These views, I presume, are but natural to those people who have worked hard to develop a praiseworthy State society. They want the credit for their work and should have it, and I believe that it is better that they should be let alone and not asked to merge their identity or work with that of another organization. My recommendation, therefore, for the future is that this committee shall do what it can to inspire the formation of State conservation of vision organizations, either as independent medical and lay societies, or as commissions of the State governments. It should stand ready to advise and assist such work in any possible manner, but it must not suggest a formal connection of any description with the committee of the American Medical Association. This committee should also be equally ready to advise or assist State organizations that already exist, and should co-operate with them in any way they may properly request.

5. I should recommend that the work of this committee be extended, so that it will become a potent power in the community. It should have a central office with a paid, interested, intelligent, energetic secretary, who could and would from time to time journey from State to State upon request, to assist in the formation and perpetuation of State societies for the conservation of vision. His traveling expenses should be paid by those who summon him to the various places. He should have a well equipped office, with one or two stenographers and a library and files, containing everything that is printed on the conservation of vision. This office should be recognized as a central bureau of assistance, advice, literature, laws, etc., where all questions concerning this subject can and will be intelligently and willingly answered. Plans should here be devised for the formation of societies; for constitutions and bylaws; for the best ways of carrying on the work; for the building of model laws; and the best means of influencing legislatures and boards of health, education, etc. The work of such an office would be enormous and its influence widespread. Its work and purposes would enlarge and develop as time progressed, and I do not see how this noble enterprise of the conservation of vision can reach its legitimate and ultimate purpose unless something of this kind is made possible. But to do this, considerable money would be absolutely necessary, and I therefore recommend that this money be forthcoming from some source and that the good work be encouraged to go on.

6. The distribution in all schools of crisply and plainly written leaflets concerning the care of eyes, ears, noses and throats. They should be taken home so that the parents can read them. They should be printed in various languages. Perhaps even a better plan would be to have suggestions of this kind printed on the blank fly leaf of all school books. The expense of printing would be almost nothing and the benefits derived would be incalculable.

I wish to thank Dr. H. B. Favill, Dr. F. R. Green, and the Council on Health and Public Instruction for the moral and financial assistance they have rendered our committee. I also wish to thank all those who have written for the Press Bulletin and the conservation of vision pamphlets. I also wish to thank all those who have assisted us in the work of the State lecture bureaus.

I shall now append a tabulated report of the State lecture bureau campaign and desire to say that a large part of the work in Ohio has been done under the auspices of the Ohio Commission for the Blind.

| No. | State | Manager | No. of lectures | No. of lecturers | No. of cities | Estimated size of audiences |
|---|---|---|---|---|---|---|
| 1 | Ga. | Dunbar Roy | 10 | 10 | 10 | 1,975 |
| 2 | S. C. | C. W. Kollock | 10 | 8 | 6 | 950 |
| 3 | Ala. | S. Ledbetter | 8 | 6 | 7 | 1,000 |
| 4 | Ore. | J. L. McCool | 3 | 1 | 1 | 300 |
| 5 | N. C. | C. W. Banner | 7 | 4 | 4 | 2,000 |
| 6 | Kans. | R. S. Magee | 7 | 5 | 6 | 1,050 |
| 7 | N. H. | E. Fritz | 8 | 7 | 8 | 1,725 |
| 8 | Wyo. | G. L. Strader | 1 | 1 | 1 | 100 |
| 9 | Tenn. | E. C. Ellett | 4 | 4 | 4 | 850 |
| 10 | N. Y. | F. P. Lewis | 2 | 2 | 2 | 225 |
| 11 | Ark. | H. Moulton | 5 | 4 | 5 | 400 |
| 12 | Mo. | C. Loeb | 15 | 12 | 8 | 2,300 |
| 13 | Utah | D. M. Lindsay | 25 | 12 | 24 | 4,675 |
| 14 | Me. | J. A. Spalding | 23 | 14 | 20 | 3,000 |
| 15 | N. D. | J. H. Rindlaub | 19 | 8 | 19 | 4,000 |
| 16 | Conn. | G. H. Warner | 2 | 1 | 2 | 850 |
| 17 | Wash. | R. W. Perry | 13 | 6 | 10 | 1,520 |
| 18 | Md. | H. Woods | 14 | 7 | 7 | 8,050 |
| 19 | Ohio | W. E. Bruner | 59 | 39 | 59 | 14,300 |
| 20 | Ky. | J. A. Stucky | 35 | 2 | 35 | 10,350 |
| 21 | Del. | J. A. Ellegood | 3 | 2 | 3 | 675 |
| 22 | Idaho | R. L. Nourse | 2 | 1 | 1 | 525 |
| 23 | Va. | J. A. White | 4 | 4 | 2 | 500 |
| 24 | Minn. | F. C. Todd | 3 | 3 | 3 | 500 |
| 25 | Iowa | E. R. Lewis | 3 | 3 | 3 | 500 |
| 26 | Wis. | N. M. Black | 1 | 1 | 1 | 250 |
| 27 | D. C. | O. Wilkinson | 2 | 1 | 2 | 200 |
| 28 | Mich. | W. R. Parker | 4 | 1 | 3 | 1,430 |
| 29 | Ind. | A. E. Bulson, Jr. | 45 | 20 | 25 | 5,000 |
| 30 | Neb. | J. M. Patton | 2 | 1 | 2 | 225 |
| | | | 339 | 190 | 283 | 69,425 |

It will thus be seen that thirty States have reported work; 339 lectures have been delivered, by 190 lecturers in 283 cities, to audiences aggregating 69,425 in numbers. When to this is added the hundreds of thousands of people who have been reached through the newspapers, by the distribution of the Press Bulletin, and the thousands of people who have been reached by the conservation of vision pamphlets, it will be seen that the work of this committee has not been in vain. I desire to add also that probably through the agency of the lectures in the various States, school inspection of eyes, ears, noses and throats, has been established in at least 200 cities.

7 West Madison Street. —New York Medical Journal.

---

### THE THERAPEUTIC VALUE OF FISCHER'S SOLUTION.

By Rufus Southworth, A. M., M. D. Assistant Professor of Therapeutics at the Medical Department of the University of Cincinnati.
CINCINNATI.

In this day and age when the doctrine of the "strenuous life" holds such complete sway, it is not surprising that the medical profession should be called upon more and more to come to the relief of those organs whose prominent function is the removal of waste products from the body; and this is true not only of the intestinal tract, but especially of the structures concerned with the elimination of the debris resulting from the metabolism of the nitrogenous or proteid elements. Thus the prefession has come to recognize the long series of symptoms grouped generically under the term "autointoxication," as requiring a thorough and continued cleansing of the intestinal tract as the basis of treatment; similarly the importance of the role played by the skin as an organ of excretion has led to the devising of many means, both mechanical and medicinal, for increasing the activity of the sweat glands; but it has been only within a comparatively short time that it has been possible with any degree of certainty or safety to excite the excretory function of the kidney to greater activity.

Attempts have been made since the beginning of medical history to produce an increase in the excretion of urine by the use of medicinal agents, and the very number and variety of the diuretics found in the materia medica gives eloquent testimony to their inefficiency. Thus the treatment, especially in acute nephritis, has resolved itself into an effort to make the skin and the intestinal tract assume, as far as possible, the duties that are naturally performed by the kidneys.

During these years which have brought an increase in the demands on the excretory organs, the brunt of the battle has fallen on the kidneys, with the result that renal insufficiency in one form or another is becoming more and more frequent, and presents a complication always serious and frequently fatal to many a condition not in itself necessarily dangerous. Thus it naturally results that any therapeutic measure which aids the kidneys in resuming their normal function finds a field of usefulness in a large number of diseases which vary widely in their pathology, but which have in common this element of renal insufficiency.

Such an agent has been placed in the hands of the profession by Dr. Martin H. Fischer, of the University of Cincinnati, in the form of a solution which bears his name, and which consists of sodium carbonate (crystallized), 10 gm., and sodium chloride, 14 gm., dissolved in one litre of distilled water. It is not within the province of this article to discuss either how or why the solution acts, but from the point of view of the therapeutist the all-important question is whether the use of this treatment increases the excretion of urine to such a degree as to relieve the symptoms, uremic and otherwise, which are attendant upon a partial or complete suppression of urine. After all, the "proof of the pudding is in the eating," and a series of cases will be described as indicating some of the conditions in which this form of treatment may be usefully employed.

Persistent Vomiting of Pregnancy.—In the first case to be discussed, the use of Fischer's solution was not employed with any idea of affecting the urinary excretion, but it occurred to the writer that it would make an excellent vehicle in which to carry other medicinal agents, should it be found desirable to give rectal medication in such cases where it was impossible or inadvisable to give remedies either by mouth or hypodermatically.

The opportunity to put this conclusion to a practical test came when the writer was called to the bedside of a patient suffering with persistent vomiting of pregnancy. She was in the third month of the uterine gestation of her first pregnancy, twenty years of age, and of a highly neurotic temperament. Vomiting had begun some three weeks earlier, and had become progressively more frequent and severe until at the time when she was first seen she had retained nothing but a little cracked ice for a period of nearly sixty hours. She suffered acutely from thirst, prostration and a sharp gnawing hunger, but the sight of food, or even its slightest odor, brought on immediate vomiting; and the vomitus finally became streaked with bright red blood.

Various measures, such as cerium oxalate, bismuth subnitrate, cocaine hydrochlorate, internally; ice caps applied to the epigastrium, etc., externally, were employed to relieve this condition but without result. Finally, on the evening of the fourth day sodium bromide, gr, xx, was dissolved in one pint of Fischer's solution and given per rectum by the drop method, about two hours being employed in giving the sixteen ounces; the thirst was at once relieved and the patient dropped into a deep sleep, awakening in the morning much refreshed; another twenty grains of the bromide in a pint of Fischer's solution was given by the same method. At noon a teacup of bouillon with a half a slice of plain toast was given by mouth; it was greatly relished by the patient and produced no nausea. A larger quantity of the same nourishment was given at 4:00 and 8:00 p. m., with no ill effect. The next day she ate her usual breakfast, dinner and supper, and neither then nor at any subsequent period of her pregnancy did she have a return of either nausea or vomiting.

In a case to be described later on reference will be made to the giving of strychnine sulphate dissolved in Fische's solution, and in that case the action of the drug as affecting the circulation was plainly perceptible; now, both bromides and alkaloids are supposed to be precipitated by alkalies, and hence to be incompatible, but in neither of these cases did precipitation occur as far as could be observed, and in both the action was prompt and satisfactory.

While the writer has not made the attempt, it should be possible to give any of the soluble iodides with Fischer's solution, especially in such cases where they are not well borne by the stomach. In fact, the use of Fischer's solution as a vehicle would only seem to be limited by its ability to hold the desired agent in solution.

Chronic Nephritis.—The second case deals with a patient who had had a chronic nephritis for a period of nearly ten years, showing itself by the presence of granular and hyaline casts in the urine and also by the finding, from time to time, of albumin in small amounts.

After a number of days of great mental and physical exertion, during which time the excretion of urine had been scanty, probably between fifteen to twenty ounces, per day, the patient developed uremic symptoms. Prominent among these were a heavy urinous odor of the breath; semi-unconsciousness from which he could be but partially aroused, and pulmonary edema; associated with an hemiplegia involving the left side, interfering with articulation and making deglutition difficult. In consultation with Dr. F. H. Lamb, efforts were at once directed toward inducing the kidneys to resume the excretion of urine, of which there had been none for a period of eighteen hours. With this object in view, and also to open the bowels, citrated caffeine, gr. iii, and calomel, gr. ½, were given at four and two-hour intervals, respectively. At the same time, one drachm of the saline ingredients of Fischer's solution dissolved in water was slowly given by mouth every two hours. This treatand the following amounts of urine were voided: 1:00 P. M. ment was instituted at nine in the morning of the first day, (same day), 12 oz.; 9:20 P. M., 12 oz.; 11:30 P. M., 16 oz.; 1:40 A. M. (following day), 4 oz.; 3:40 A. M., 3 oz.; 8:30 P. M., 8 oz.

At this point the calomel was discontinued, and rubinat was given at 7:00 A. M. and at noon. The bowels began to move at 1:45 P. M., and moved five times up to 6:20 P. M., probably there was some urine voided during that time but not in any great amount. An examination showed much albumin and casts of every description.

At this point, as the Fischer's solution was not being well borne by the stomach, and as swallowing was becoming more and more difficult, it was discontinued by mouth and given by the drop method per rectum for a period of two hours, with two-hour intervals. At midnight a considerable quantity of urine was passed involuntarily, followed by: 3:00A. M., 8 oz.; 7:00 A. M., involuntarily; 8:30 A. M., 8 oz.

Thus, at the beginning of the third day the kidneys showed evidence of increase in their activity, otherwise the patient showed no improvement, and 1:00 P. M. found the temperature 101.4° F., by axilla, pulse 112, respirations 34, and the pulmonary edema was increasing. Adrenalin chloride, 1 to 1,000 was given in five-drop doses by mouth every two hours, and the citrated caffeine was reduced to two grains. During this twenty-four hour period, thirty-four ounces of urine were voided, the albumin and casts were much reduced, and the fourth day showed improvement in the general symptoms. The solution was continued either by rectum or mouth, and the amount of urine increased from day to day until by the eighth day fifty-six ounces were passed, with but little albumin and few casts. Convalescence was slow, but eventually the patient was able to resume his usual duties.

TYPHOID FEVER.—Every physician in general practice is familiar with the importance of the part played by renal insufficiency in those patients who are suffering from acute diseases of any kind; also that such a complication is not only liable, but is almost certain to occur in that class known as "chronic alcoholics," and that the appearance of uremic symptoms at once makes the prognosis exceedingly grave. Therefore, any form of treatment which aids the kidneys in performing their normal function should have a marked effect by either lessening or entirely eliminating this dangerous complication, and thus giving the patient a considerably better chance of ultimate recovery.

With this object in view, Fischer's solution was used under the following circumstances in a case of typhoid fever in a male patient, thirty-five years old, who was known to have been a steady drinker for some fifteen years, and who had been under treatment at various times previous to this sickness f°r an alcoholic gastritis, and had also been threatened more than once with delirium tremens.

The onset and course of the disease during the first ten days did not differ materially from the picture which typhoid commonly presents, but at the end of the second week the urine became scanty, an active delirium appeared, which was qiuckly followed by coma. On the thirteenth day the patient passed but eight ounces of urine, which was highly colored and smoky; an examination showed that the specimen contained albumin and was loaded with casts, chiefly epithelial and granular. At this point Fischer's solution was given per rectum by the drop method, being used continuously for four hours. It was then withdrawn for a period of two hours and then resumed for four hours; in this manner the solution was employed for two days.

During the first twenty-four hours the amount of urine was increased, probably about twenty ounces being passed, most of which, however, was voided involuntarily. The next twenty-four hours showed a still further increase, and on the third day the patient regained consciousness and the amount of urine for the day was in the neighborhood of forty ounces; his mind was perfectly clear, but on the next day he had several attacks of syncope, due to an acute dilation of the heart superimposed upon a chronic myocarditis, from which the patient succumbed a few days later.

In this case there would seem to be no doubt but that the use of this solution had a most decided effect in relieving the uremic condition and the symptoms attendant upon it, and should be of great value in treating the acute illness of the chronic alcoholics, especially diseases which run a less protracted course, as pneumonia. It is also entirely possible that if used early it might prevent the onset of delirium tremens as a complication of severe injuries when inflicted on this type of patient.

CHRONIC MYOCARDITIS.—The fourth case in which Fischer's solution was used, was for a patient in whom the primary trouble was a chronic myocarditis, plus a mitral regurgitation, associated with a moderate degree of arteriosclerosis. She was seventy years of age, and had had attacks of myocardial insufficiently of moderate severity for at least two years previous to the time that she was first seen by the writer. On that occasion an examination showed edema of the lower extremities, extending to the knees, ascites, no edema of the hands, arms or face, lips and finger nails somewhat cyanotic, considerable dyspnea with pulmonary edema, causing almost constant coughing, the pulse was weak and irregular, a cardiac murmur was heard at the apex and transmitted to the axilla, no temperature, and the bowels open; between thirty and forty ounces of urine was passed in twenty-four hours, which showed no especial abnormality; patient complained of nausea, but had no active vomiting; sat in a chair and moaned continuously but complained of no acute pain.

Treatment was instituted at once in the form of tincture of strophanthus, m. vii, adrenaline chloride, 1 to 1,000 m., viii, and digitaline, gr. 1-30, plus citrated caffeine gr. ½, given by mouth at six-hour intervals, being four doses in the twenty-four hours.

Within two days there was noticeable improvement, the coughing and dyspnea, the pulse was more regular and stronger,
coughing and dyspnea, the pulse was more regular and stronger, at this point the patient had a convulsion of terrific severity, the lips and face became black, great quantities of black fluid gushed from nose and mouth, every muscle in the body was rigid, the eyes wide open and the pupils fully dilated; suddenly all the muscles relaxed, the chin fell forward and the head back upon the pillow, and to all appearances the patient was dead; however, in a few moments a very shallow respiration was resumed.

Into the already opened vein one and a half pints of Fischer's solution was given, requiring some thirty minutes for its introduction; a very slight convulsion occurred just as the incision in the arm was being closed and that was the last convulsion from which she suffered, the respiration became deeper and the cyanosis was less marked. The solution was given per rectum all night, the amount of urine, which was voided involuntarily, was increased; eighteen hours later the patient regained consciousness and during this time the solution was given per rectum at frequent intervals, convalescence was rapid though albumin was found in the urine in diminishing amounts for two months. Today when more than a year has elapsed she has no trace of albumin and seems to be perfectly well.

Mention must be made of the aid given by Miss Licking, a graduate nurse of the Jewish Hospital, but for whose great skill and untiring devotion to the patient a successful outcome could not have resulted.—The Lancet-Clinic.

---

### SIXTEENTH ANNUAL MEETING AMERICAN PROCTOLOGIC SOCIETY.

(Continued.)

### CRUDE AND CARELESS DIAGNOSTIC METHODS, AND RESULTS OF SAME, IN SOME RECTO-COLONIC CONDITIONS. By Jno. L. Jelks, M. D., of Memphis, Tenn.

The author criticizes the busy doctor and surgeon who too hastily yields to a conclusion and treats recto-colonic diseases without sufficient investigation to warrant or obtain a correct diagnosis.

Reference is made to cases operated on for appendicitis, which disease may be an extension and inflammation originating in the rectum or colon.

Cases are cited to show the frequency and at all times the liability of mistaking a condition for an infection or ulceration of the colon, specific in character, when a coloptosis or pericolic membranes, or both, were the true etiologic factors. Stress is laid on the importance of urinalysis, microscopic examinations and the X-ray in recto-colonic cases.

A harder nodular calcareous degeneration of the outer zone of the mamma has been observed as a sequence of coloptosis and defective drainage. In another case, in which was found a cecum cradled in pericolic membranes, and a coloptosis, a duodenal ulcer was diagnosticated. In this case the urinalysis, the history, and general toxic appearance of the patient, pointed to true etiology.

Case reports are given in which diarrhea was the dominant symptom, though impaction, pericolic membranes, and ptosis were the true etiology.

The author calls attention to his prior reference to, and work of establishing the importance of conserving the ilio-cecal valve; also to the syphonage of a ptosed colon after short circuiting operations, which he accomplished by a second anastomosis between the blind colon and the sigmoid or rectum below the first anastomosis.

Importance is claimed for a microscopic examination of the intestinal contents of patients who suffer from attacks of appendicitis, and of the contents of the removed appendix; and the author insists that in the event that pathogenic amebae are found appendico-cecostomy should be performed instead of appendectomy.

The author refers to his observation of quite marked congestion of blood in the visceral vessels themselves in these cases of ptosis and defective intestinal drainage.

The author refers to the frequency with which he encounters cases of inoperable cancers of the rectum and intestines, the neglect of which is most often due to the fear of examination of those suffering with symptoms in the regions referred to.

Reference is made to the operation of appendico-cecostomy as being practically free of danger to life. In his opinion this operation would save almost every life that is to-day caused by the ravages of amebic colitis.

---

### ABSCESS ORIGINATING IN A PILO-NIDAL SINUS.

By Louis J. Krouse, M. D., of Cincinnati, Ohio.

The writer states that a pilo-nidal sinus is a congenital defect due to a faulty development of the foetus. It is usually located in the median line over the coccyx or the sacrum. Inflammation developing in the sinus is followed by burrowing of pus into the neighboring tissue. Inflammation of this sinus must be differentiated from necrosis affecting the sacral or coccygeal bone; from abscess originating in the sebaceous gland of this region; and from true fistula-in-ano. The treatment consists in the complete obliteration of the walls of the sinus.

### ABNORMALITIES OF THE COLON, AS SEEN WITH THE ROENTGEN RAY: LANTERN SLIDE DEMONSTRATION.

By W. I. LeFevre, M. D., of Cleveland, Ohio.

The entire alimentary tract can now be successfully examined with the X-ray, some parts more readily and successfully than others, according to the degree of satisfaction arranging themselves in the following order: Colon, Stomach, Small Intestine. Two methods of examination are used. First, Roentgenoscopy, which is the examination with the fluoroscope. Second, Roentgenography, the making of X-ray plates. The Colon is also accessible from either end, that is, it can be examined by following the bismuth meal through from the stomach, or by giving an opaque enema of bismuth sulphate. In the former method the motor phenomena of the colon can be observed, in the latter, the size, position and contour can be seen.

### A MUCH NEEDED INNOVATION.

So many accidents have occurred through mistaking mercury bichloride tablets for those intended for headaches, insomnia, indigestion, etc., that the necessity for some distinctive feature in shape, color or other means be adopted by manufacturers that may effectually remedy the evil. Numerous devices, including shape, color and style of container have been tried, with only partial success; for, more often than otherwise, the corrosive sublimate tablets have been hurriedly taken at night from the medicine chest or shelf, from containers closely resembling other practically harmless ones with such self assurances of security, while in order not to disturb others, and without light to confirm the identity of the tablet, deplorable accidents have been all too frequent. Under such circumstances in the hurry of the moment, color counts for nothing, while shape of tablet or container alone is practically negligible.

Recently an ingenious device has been worked out by Messrs. Sharp & Dohme, whereby these highly poisonous mercury bichloride tablets are THREADED; attached to one another by a thread; the thread to be cut in order to secure a tablet for use. This procedure necessarily calls for light and cutting the thread, thus centering the mind upon the act. No other tablet than the mercury bichloride will be thus marketed threaded. This unique feature, together with a clover shape, and distinctive color of the tablet, blue; the trefoil (or clover) shape with the word poison plainly stamped on each, and the peculiar shape of the bottle container, which will rivet the attention of the person immediately upon taking it in the hand, will, it is confidently believed, entirely obviate the possibility of accidents.

The Threaded Mercury Bichloride Tablets are made only in one color, blue, and offered in containers of twenty-five tablets each. They will not be supplied in bulk. Physicians should, therefore, prescribe them in the original containers.

